# Marine bioactive peptides as potential therapeutic agents for wound healing – a review

**DOI:** 10.1080/07853890.2025.2530693

**Published:** 2025-07-13

**Authors:** Kai Zheng, Ziwei Yang, Te Ba

**Affiliations:** a Department of Burn Surgery, The Third Affiliated Hospital of Inner Mongolia Medical University (Inner Mongolia Bao Gang Hospital), Baotou, China; b Department of Pediatric Surgery, Tianjin Medical University General Hospital, Tianjin Medical University Graduate School, Tianjin, China

**Keywords:** Wound healing, skin injuries, marine peptides, antimicrobial peptides, ceRNA network

## Abstract

**Context:**

Skin wounds resulting from dermatological diseases and other specific causes present significant challenges in health care. These wounds can lead to infections, chronic conditions, long-term complications and even mortality. Consequently, developing highly effective and viable therapies for wound healing is important. Marine bioactive peptides are increasingly being utilized because of their favourable biological properties, which include anti-inflammatory, antioxidant, antimicrobial, antiageing, antitumour and antihypertensive effects. These peptides offer several advantages, including high efficacy, excellent safety, clear target specificity and low allergenicity. Notably, scientific evidence has demonstrated that marine bioactive peptides promote skin injures repair and has underscored their substantial potential in wound healing.

**Objective:**

Our objective is to provide molecular insights that may facilitate the development of innovative therapeutic agents aimed at improving wound healing and skin repair.

**Materials & methods:**

This review synthesizes information from major databases, including ScienceDirect, Google Scholar, Scopus, PubMed and Springer Link. Publications were selected without date restrictions and used terms such as skin injures, wound healing, marine bioactive peptides, antimicrobial, ceRNA. Articles related to agriculture, ecology, or synthetic work or those published in languages other than English were excluded.

**Results and conclusions:**

In this review, we integrated and examined the current understanding of wound care. Additionally, we analysed the underlying molecular mechanisms involved to elucidate the characteristics of marine bioactive peptides.

## 1. Introduction

Wounds are categorized into two types based on the basis of duration and healing process: acute and chronic. Acute wounds caused by extreme temperature changes, chemical exposure or radiation exposure are further classified into various types based on their size and depth, including superficial, deep dermal, or full thickness wounds. The acute wounds typically heal within 4 to 12 weeks, restoring both functional and anatomical skin integrity [1]. The dysregulated reparative processes due to certain factors can manifest as either an entirely failure heal or excessive scarring. Specifically, wounds that do not heal in 12 weeks are categorized as chronic wounds [2]. Chronic wounds can be further subdivided into diabetic ulcers, pressure ulcers and arterial/venous ulcers. Both ageing and diabetes can result in the changes of skin tissue mechanics, progressive loss of the dermal matrix, reduced resilience and increased vulnerability to friction damage [3]. Therefore, due to reduced hydration, altered skin and atrophy, the skin of these individuals is more prone to injury. The infection caused by pathogenic microorganisms can result in the high levels of inflammation and oxidative stress. Cellular senescence is widely implicated as a contributing factor in the pathology of aged and diabetic wounds. The chronic wound environment provides an ideal platform for the senescent cells, which prolongs the inflammatory phase [4]. Improved clinical management could more effectively prevent some of these wounds. However, many patients still do not respond well to current treatment, which emphasizes the importance of gaining a deeper understanding of the mechanisms involved in wound healing.

Currently, there is surging interest in marine bioactive peptides because of widespread presence and potent biological activity. Marine bioactive peptides are being utilized due to their beneficial biological properties. These properties include anti-inflammatory [5], antioxidant [6], antimicrobial [7], antiageing [8], antitumour activities [9] and antihypertensive [10]. They are primarily derived from sources such as fish, crustaceans, mollusks, algae and specific marine by-products including fish skin, shells, muscles and offal [11,12]. These marine bioactive peptides offer advantages such as high activity, good safety, clear specificity of the target and low allergenicity [13]. Notably, marine bioactive peptides have been proven to facilitate skin tissue repair and demonstrate potential in wound healing.

In this review, we summarized the physiological mechanisms involved in wound healing. We also explored how understanding the interactions between marine bioactive peptides and tissue can promote wound healing. Furthermore, we emphasized the correlation between marine bioactive peptides and clinical outcomes, which can inform future therapeutic interventions aimed at enhancing wound healing.

## 2. Skin injury and wound repair pathophysiology

### 2.1. Acute wound healing

**Figure 1.**
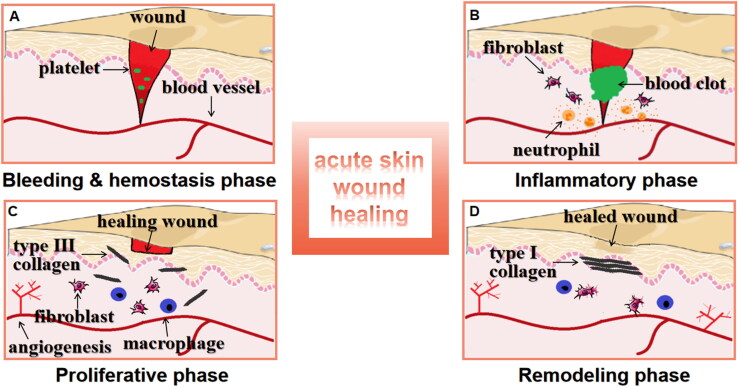
The stages of acute skin wound healing. (A) The bleeding and haemostasis phase: after vascular damage, platelets begin secreting to stop bleeding and forming a scab. (B) The inflammatory phase: blood clot regularly shape in one to three days after damage. Inflammatory cells like neutrophils and fibroblasts are recruited to the wound. Epidermal cells occur migration. (C) The proliferative phase: macrophage phagocytosis removes bacteria, dead cells and foreign object. Angiogenesis begins developing. Fibroblasts in the vicinity of wound induce the ECM deposition. (D) The remodelling phase: wound begins contracting, the collagen III is replaced with collagen I, formation of a rich collagen scar is followed.

Wound repair is a natural physiological response to tissue injury that has occurred; the body has developed efficient mechanisms to close breaches in the skin barrier. It is specifically designed to restore tissue integrity and prevent infection. This process involves several phases: bleeding and haemostasis, inflammation, proliferation (formation of new tissue) and remodelling [14] (Figure 1). The process heavily relies on the interactions between different skin compartments, the extracellular matrix (ECM) and systemic contributions. The communication between these variables is facilitated by the expression of adhesive molecules, growth factors, cytokines and chemokines. These include platelet-derived growth factor (PDGF), platelet activity factor (PAF), nitric oxide (NO), interleukins (IL-1, IL-6) and tumour necrosis factor-alpha (TNF-α), etc. They play a critical role in facilitating interaction and communication among the variables [15].

#### 2.1.1. One to three days after injury

##### 2.1.1.1. Haemostasis and humoral inflammation

Bleeding can be rapidly controlled through vasoconstriction and the activation of the coagulation cascade. A few minutes after injury, platelets in the bloodstream adhere to the injury site and activate upon exposure to subendothelial collagen. Together with thrombin and fibronectin, they coagulate to form a clot [16,17]. The clot is primarily composed of crosslinked fibrin, plasma fibronectin and ECM proteins [18]. It releases factors such as PDGF, tumour growth factor (TGF)- α, PAF, fibronectin and serotonin [19]. This process initiates humoral inflammation.

##### 2.1.1.2. Cellular inflammation

Vasodilation and increased vascular permeability subsequently lead neutrophils, monocytes, lymphocytes and fibroblasts to migrate [20]. Neutrophils are the initial subset of immune cells that respond to tissue injury. They utilize their antibacterial properties to phagocytose and eliminate contaminating microorganisms, while also secreting various proteases and reactive oxygen species (ROS) that attract additional neutrophils and macrophages [21]. Approximately 48 to 96 h following tissue injury, monocytes are the second type of cells to increase during the inflammatory phase. These monocytes then differentiate into macrophages [22]. With the assistance of transforming growth factor- β (TGF- β), neutrophils are rapidly phagocytosed by monocyte-derived macrophages after they eliminate devitalized host tissue and infectious agents, unless the neutrophils still pose a threat. Additionally, tissue-resident macrophages are capable of degrading and removing injured tissue debris [23]. These macrophages demonstrate a pro-inflammatory M1 phenotype, which results in the production of IL-1, TNF and ROS. Subsequently, they undergo a transition into an anti-inflammatory M2 profile. This transition leads to the production of IL-10, epidermal growth factor (EGF), fibroblast growth factor (FGF), vascular endothelial growth factor (VEGF), TGF- β and matrix metalloproteinase (MMP) [24]. The VEGF, FGF and TNF are involved in mediating angiogenesis. Additionally, EGF, TGF- β, IL-1 and TNF play roles in regulating matrix synthesis. Furthermore, TNF promotes the expression of MMP by fibroblasts, monocytes and macrophages, which further clears damaged ECM [25].

#### 2.1.2. Four to 10 days after injury

##### 2.1.2.1. New tissue formation

The proliferative phase of healing involves activating fibroblasts, macrophages, endothelial cells and keratinocytes to close wounds, deposit matrix and promote angiogenesis. The growth factors, particularly TGF- β, stimulate fibroblasts to proliferate and secrete components of the ECM such as collagen (mechanical strength to the wound), proteoglycans, fibronectin and hyaluronic acid [26]. By producing proteases such as MMPsng angiogenesis, macrophages degrade the dense fibrin network and drive endothelial migration through the release of chemotactic factors including TNF- α, VEGF and TGF- β [27]. Angiogenesis mediated by endothelial cells plays a crucial role in providing oxygen and nutrient supply to the cells [28]. These vessels vascularize the ECM by promoting fibroblast proliferation, leading to the formation of highly vascularized granulation tissue. This tissue tends to degrade the existing blood clot [29]. As early as 12 h post-injury, keratinocytes initiate partial epithelial–mesenchymal transition, a process that is stimulated by keratinocyte growth factor 1, which is primarily produced by fibroblasts [30]. The differentiation and proliferation of keratinocytes lead to re-epithelialization, which promotes the proliferation and migration of epithelial cells from wound borders. This creates a superficial epidermal cover for the wound.

#### 2.1.3. Ten days to two weeks after injury

##### 2.1.3.1. Tissue remodelling

The apoptosis of myofibroblasts, macrophages and endothelial cells signifies cessation of contraction, the resolution of inflammation and involvement of vascularization respectively [31–33]. Under the regulation of MMPs and their inhibitors, wound tissue predominantly contains collagen type I in the ECM, which replaces collagen type III [34]. Ultimately, the scar will develop as dense connective tissue with reduced tensile strength and elasticity, which is attributed to alterations in the composition of the ECM [35]. Granulation tissue is replaced by acellular scar tissue following the completion of wound repair and the apoptosis of myofibroblasts [36].

### 2.2. Chronic wound healing

**Figure 2.**
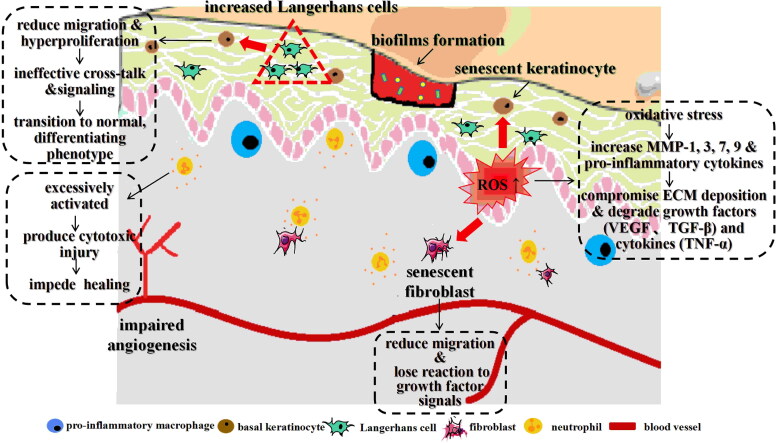
The stages of chronic skin wound healing. The pro-inflammatory macrophages, Langerhans cells significantly increase in chronic wounds. Meanwhile neutrophils are excessively activated, to produce cytotoxic injury and impede the wound healing. The increase of Langerhans cells make keratinocytes cease to proliferate and transit to a normal, differentiating phenotype. ROS can impair the chronic wound healing by inducing MMPs (MMP-1, 3, 7, 9) and prolonging secretion of pro-inflammatory cytokines. The MMPs are responsible for compromising ECM deposition and degrading growth factors (e.g. VEGF and TGF-β) and cytokines (e.g. TNF-α). Consequently, elevated ROS can produce highly senescent dermal fibroblasts and keratinocytes by activating proteolysis, showing reduced migratory capacity and unresponsiveness to growth factor signals.

Chronic, persistent wounds generally stagnate between the inflammatory and proliferation stages. They fail to rebuild the wound tissue architecture because of ongoing tissue destruction (Figure 2). Chronic wounds exhibit a significant rise in neutrophils [37], pro-inflammatory macrophages [38], Langerhans cells [39], IL-1β and proteases [40,41], linked to clinical ulcer severity [42]. Neutrophils are excessively activated to produce cytotoxic injury and impede the wound healing [43]. In diabetes, high glucose levels cause macrophages to function improperly, excessive activation of NLRP3 inflammasome promotes increased expression of IL-1β, which subsequently leads to elevated production of pro-inflammatory cytokines and exhibits an increased presence of pro-inflammatory macrophages [44]. Meanwhile, due to the increased presence of Langerhans cells, keratinocytes cease to proliferate and transform into a normal, differentiating phenotype [45–48]. Under the ROS- mediated transcription, oxidative stress can impair the chronic wound healing by inducing MMPs (MMP-1, 3, 7, 9) and prolonging secretion of pro-inflammatory cytokines. Due to their higher protease activity, the MMPs are responsible for compromising ECM deposition and degrading growth factors (e.g. VEGF and TGF- β) and cytokines (e.g. TNF- α) [49,50]. Consequently, elevated ROS level can produce high levels of senescence in dermal fibroblasts and keratinocytes by activating proteolysis [51,52]. Nonhealing ulcers show markedly decreased levels of the TGF-β receptors and downstream signalling cascade components. Additionally, the fibroblasts within the ulcer exhibit signs of senescence, displaying reduced migratory capacity and decreased responsiveness to growth factor signals [53,54].

When the skin is disrupted, there is an imbalance between the natural and pathogenic microbiota, increasing the risk of infection (primarily *Staphylococcus aureus*) [55]. In diabetic mice, macrophages near the abscess produced elevated levels of leukotriene B4 (LTB4) [56]. This inflammatory lipid mediator directs the infiltration of neutrophils and contributes to the formation of a structured abscess [57,58]. In the absence of commensal microbiota, high levels of TNF- α and IL-10 likely contribute to elevated levels of VEGF, type III collagen and TGF- β. This promotes angiogenesis at wound sites, accelerating skin wound healing and partially reducing scarring. Pathogenic microbiota colonize the wound and irreversibly adhere to the wound surface, forming intricate community structures of bacteria commonly, usually referred to as biofilms [59]. The primary constituent of biofilms is the extracellular polymeric substance (EPS), which consists of polysaccharides, proteins, glycolipids and DNA [60–63]. Biofilms can form within 10 h after-injury in at least 60% of all chronic wounds [64,65]. Persistent inflammation is fuelled by chronic wound infections, creating a cycle of infection, inflammation and poor healing.

## 3. The mechanism of marine bioactive peptides in promoting wound healing

An increasing number of marine bioactive peptides have been described regarding their potential in wound healing [66]. Marine bioactive peptides are typically found in encrypted forms within the structure of a parent protein. These peptides are produced and released through chemical hydrolysis or enzymatic intervention [67]. Therefore, they may offer a new framework for the development of effective wound-healing medications (Table 1).

**Table 1.** Potential marine bioactive peptides.

Species	Peptide	Sequence (amino acid /Genbank)	Mechanism of action	Reference
Orange-spotted grouper	Epinecidin-1	AY294407.1 (Genbank)	Antimicrobial against *Vibrio harveyi*-infected Grouper decrease of the negative surface charge of bacteria, resulting in membrane distortion, bacterial death applied to sensitive and antibiotic-resistant, antimicrobial activity against methicillin-resistant *Staphylococcus aureus*, stimulation epithelial cell proliferation and migration, decrease of marker C-reactive protein (CRP) and IL-6 in the wound area, promotion the deposition of collagen in wound vicinity; Epinecidin-1 antimicrobial activity has been evaluated and proved on 24 bacterial, six fungal and one protozoan strains	[69]
*Porphyra yezoensis*	TPDSEAL	Thr-Pro-Asp-Ser-Glu-Ala-Leu	Antimicrobial activity against *S. aureus*; damage of the cell wall and membrane and enhancement the permeability of cells, the outflow of intracellular substances and death of bacteria	[70]
*Octopus minor*	Octominin	GWLIRGAIHAGKAIHGLIHRRRH	Membrane damage to the *Candida albicans* cells, decrease of cell viability and increase of ROS level	[71]
*Olivancillaria hiatula*	Peptide mix	–	Inhibition biofilm formation by interfering with cell-to-cell communication in *Pseudomonas aeruginosa*; requiring ≤39 μg/mL to inhibit 50% biofilm formation; biofilm inhibition at 1/2 MIC whereas 2.5 mg/mL was required to degrade preformed biofilm. At 1/2 MIC of peptide, the expression of pyoverdine was inhibited by 72%	[72]
Green tiger shrimp (*P. semisulcatus*)	*P. semisulcatus* antimicrobial peptides crustin	- (Cysteine rich AMP with a molecular weight of 7–14 kDa, cationic, whey acidic protein (WAP) domain at the carboxyl terminus and amphipathic secondary structure)	Biofilm inhibition and eradication (antibacterial activity against Gram positive strains *B. thuringienisis* and *B. pumilis*);	[73]
*Mytilus coruscus*	Myticusin-β	–	Antimicrobial activity against gram-positive (*Bacillus cereus, Bacillus subtilis, Clostridium perfringens, S. aureus, Streptococcus iniae, Streptococcus mutan*s) and gram-negative bacteria (*Escherichia coli, P. aeruginosa, Vibrio alginolyticus, Klebsiella pneumoniae*), anti-parasitic activity	[76]
*Octopus vulgaris* (suckers)	OctoPartenopin	AGTNK	Antibacterial activity against *S. aureus*, *P. aeruginosa* and a yeast *C. albicans*, biofilm inhibition and eradication	[77]
Half-fin anchovy	HAHp2-3-I	MLTTPPHAKYVLQW, SHAATKAPPKNGNY, PTAGVANALQHA, QLGTHSAQPVPFFW, VNVDERWRKL, LATVSVGAVELCY, NPEFLASGDHLDNLQ, PEVVYECLHW	Adherence to the bilayer surface of the membrane in *E. coli* through electrostatic effects and accumulation, the bilayer undergoes irreversible damage	[84]
*Scylla paramamosain*	Scyreptin_1–30_	MH488960 (Genbank)	Disruption bacterial membranes, reduction in bacterial load and causing bacterial death; exhibition of a broad-spectrum antimicrobial activity; demonstration of significant potency against both bacteria and fungi and even against the clinically isolated multidrug-resistant bacteria *P. aeruginosa*; potentially serving as a topical skin treatment, including those caused by *P. aeruginosa*.	[86]
Nile tilapia (*Oreochromis niloticus*)	Tilapia piscidin 4	FFRHLFRGAKAIFRGARQGXRAHKVVSRYRNRDVPETDNNQEEP	Kill of *Vibrio vulnificus* and *P. aeruginosa* due to conformational differences of the peptide when binding to host membranes, inducement of epithelial cells proliferation, promotion of collagen I and III, keratinocyte growth factor, keratin 10 and enhancement of wound healing activities mediated by EGF, TGF and VEGF	[90,98]
*Boleophthalmus pectinirostris*	Bolespleenin_334–347_	–	Kill of *Acinetobacter baumannii* and *S. aureus* by disrupting the structural integrity of the bacterial membrane, leading to leakage of the cellular contents and inducing accumulation of bacterial endogenous ROS, inhibition of biofilm formation of *A. baumannii* and *S. aureus* and long-term treatment did not lead to the development of resistance, reduction in bacterial load and more favorable wound healing.	[78]
*Sipunculus nudus*	SNCP	Arg -Leu-Tyr -Pro	Regulation of collagen formation and remodelling: enhancing collagen deposition and preventing the pathological effects caused by the TGF-β/Smads pathway, provision of nutritional support for wound healing with its essential amino acids	[100]
Atlantic salmon (*Salmo salar*)	SS-SCP	–	Decrease of proinflammatory cytokines (TNF-α, IL-6 and IL-8) and upregulation of anti-inflammatory cytokine IL-10, VEGF and β-FGF, acceleration of wound healing by controlling the inflammation and increasing angiogenesis and collagen deposition	[101]
Sea cucumber	SCCOP	Asp-Glu-Ser-His-Gly-Thr-Arg-Ala-Tyr-Cys-Val-Met-Phe	Reduction of CRP, IL-6, IL-8, TNF-α and ROS, increase of IL-10 and VEGF during the wound healing in diabetic mice	[95]
*Pinctada martensii*	SMP	–	Procoagulant activity and enhancement of wound closure, inhibition of IL-10, promotion of the secretion of fibroblasts and keratinocytes, acceleration of collagen cross-linking deposition, shortening of epithelialization time by upregulating TGF-β1 and cyclin D1	[102]

### 3.1. Marine bioactive peptides improve wound healing through antimicrobial mechanisms

When a wound is exposed to environmental factors, pathogenic microbiota disrupts the delicate balance of the wound microenvironment, thereby increasing the risk of infection. Many studies have shown that the effective antibacterial and bactericidal properties of marine antimicrobial peptides, highlighting their potential use as fungicides in wound protection. It is crucial to investigate bioactive peptides that resist a range of bacteria, such as *S. aureus, Escherichia coli, Pseudomonas aeruginosa, Enterococcus faecium* and *Klebsiella pneumoniae* [68]. They are widely observed in a variety of marine species, such as Orange-spotted grouper, *Porphyra yezoensis*, *Octopus minor*, *Mytilus coruscus*, *Olivancillaria hiatula*, Green tiger shrimp, Cyanobacteria and *Hypoptychus dybowskii* [69–76].

#### 3.1.1. The mechanism of biofilm inhibition

Bacteria are capable to generate biofilms in the infections of human tissues. The continued growth of cells within the biofilm leads to the detachment from the biofilm. Then it spreads the infection to establish new biofilms in other infection area. The pentapeptide AGTNK isolated from the octopus arms suckers exhibits significant antimicrobial activity against *P. aeruginosa* and *S. aureus*. The peptide is encoded within the sequence of calponin-2-like isoform X1 and occurs multiple times, along with some variant sequences. This results in several bioactive peptide molecules being yielded from each parental protein, which serve as inhibitors and eradicator of biofilm in the tested microorganisms [77]. The short peptides (pentapeptide) are susceptible to degradation by proteases, it requires chemical modifications or delivery systems to enhance stability, such as nanomaterial carrier. The production of parental proteins may increase the complexity of large-scale manufacturing processes. The polypeptide library characteristic improves the diversity of function. A combined antimicrobial therapy was developed based on the ‘one source, multiple peptides’ strategy to reduce the drug resistance risk of single peptide.

Bolespleenin_334–347_ from the mudskipper *Boleophthalmus pectinirostris* devastates the bacterial membrane, causing outflow of the cellular contents. It also inhibits biofilm formation and induces the generation of bacteria ROS of *S. aureus* and *Acinetobacter baumannii*. Bolespleenin_334–347_ maintained stable activity against clinically multi-drug resistant bacterial strains, reduced bacterial load and accelerate wound healing [78]. But bacteria ROS generation may pose a risk of oxidative stress in host cells, and its safety needs to be verified.

Although marine bioactive peptides with moderate MIC (minimum inhibitory concentration) values are not suitable for use as single potent antibiotics, they exist the effect on synergy with traditional antibiotics by destabilizing the biofilm inhibition.

#### 3.1.2. The mechanism of bacterial membrane disruption

Some peptides causes the saddle splay membrane curvature generation in bacterial membrane followed by vesicularization and lysis. The membrane disruption ultimately results in bacterial death. Epinecidin-1 is a naturally occurring peptide found in the orange-spotted grouper (*Epinephelus coioides*). Investigation of the *Vibrio harveyi-*infected grouper has demonstrated that the highest expression of this peptide can be observed in the skin, followed by the gills, liver and kidney [79]. Epinecidin-1 can decreases the negative surface charge of bacteria, resulting in membrane distortion [80]. When Epinecidin-1 acts on sensitive and antibiotic-resistant *P. aeruginosa*, it kills bacterial death without causing any adverse behavioural effects, hepatotoxicity, or nephrotoxicity [81]. The mechanism of physical membrane destruction may reduce the drug resistance development. The natural extraction cost of raw orange-spotted grouper is more expensive than other marine species. In the future, it is necessary to further explore its industrial production methods, human pharmacokinetics and long-term toxicity.

HAHp2-3-I is the pepsin hydrolysate derived from half-fin anchovy (HAHp), and it exhibits antibacterial activity against both Gram-positive and Gram-negative bacteria [82]. This charged peptides have the capability to assemble into elongated strands, chaotic coils, as well as intricate alpha helix structures [83]. HAHp2-3-I, with net charges, adhere to the bilayer surface of the membrane in *E. coli* through electrostatic effects and subsequently accumulate like a carpet. Ultimately, the bilayer suffers irreversible damage similar to that caused by detergents [84]. *via* the ‘carpet effect’ mode of physical membrane destruction and sterilization, it can reduce the drug resistance. The charge-dependent mechanism offers new insights for designing targeted antibacterial drugs. Based on the characteristics of alpha helix structures and charge, more stable peptides with enhanced targeting specificity can be designed. The stability of HAHp2-3-I in the multi-level cascade biological microenvironment and the development of low-cost mass production present issues that researchers need to address.

Scyreptin_1–30_, a newly discovered cationic antimicrobial peptide derived from the marine invertebrate *Scylla paramamosain*, demonstrates rapid bactericidal kinetics, excellent thermal stability and broad-spectrum antimicrobial activity [85]. This peptide disrupts bacterial membranes, reduces bacterial load and ultimately causes bacterial death. Additionally, it exhibits potent anti-biofilm activity against multidrug-resistant *P. aeruginosa* in mouse model of burn infection [86]. Thus, Scyreptin_1–30_ can be applied as an anti-infection adjuvant to prevent or treat secondary infections after burns. Meanwhile, the inhibition of biofilms formation on the surfaces of catheters and implants, as well as the reduction of hospital-acquired infections, are worth exploring. Scyreptin_1–30_ promotes the medical application of marine bioactive peptides and expands the sources of antibiotics.

Piscidin is a cationic marine antimicrobial peptide expressed by mast cells of Nile tilapia (Oreochromis niloticus) [87]. The piscidin family, including coding sequences TP1, −2, −3, −4 and −5, is characterized by an amphipathic α-helical structure, which underlies their potent bactericidal activity against a wide range of microorganisms [88]. Notably, the antimicrobial activity of Tilapia piscidin 4 (TP4) is superior to that of Epinecidin-1 [89]. TP4 exhibits greater antimicrobial activity against *Vibrio vulnificus* and *P. aeruginosa*, which is attributed to specific conformational differences when binding to bacterial membranes [90]. The unique amphipathic α-helical structure and broad spectrum of high efficiency antibacterial may exist effect on acute infection wound.

In summary, marine bioactive peptides are a vast family of natural antibacterial chemicals produced almost entirely by various marine organisms. Due to their high antibacterial activity and potential to provide an auxiliary immunoregulatory properties in certain cases, they offer a promising solution for wound healing.

### 3.2. Marine bioactive peptides improve wound healing through skin repair mechanisms

Skin wound healing remains a challenging issue despite extensive research efforts. Recently, there has been growing research emphasizing the effectiveness of marine bioactive peptides in promoting skin wound healing. This growing focus highlights the rising importance of this field (Table 1).

#### 3.2.1. The regulation of inflammation

Excessive inflammation can damage normal tissues during wound healing. This damage is the main cause of fibrosis disease. The marine antimicrobial peptide Epinecidin-1 not only exhibits potent antimicrobial activity against methicillin-resistant *S. aureus* but also promotes burn wound healing in a swine model by enhancing skin repair [91]. Notably, applying Epinecidin-1 to the wound effectively reduces the levels of C-reactive protein (CRP) and pro-inflammatory cytokine IL-6. Furthermore, Epinecidin-1 treatment promotes collagen deposition near the wound site, ultimately enhancing tissue repair [91,92]. One of the main advantages of Epinecidin-1 is its strong antimicrobial activity against multidrug-resistant pathogens. It also has some other characteristics including immunomodulatory, wound healing and antioxidant effects. These advantages will be very beneficial for clinical research.

SS-SCP is a marine collagen peptide extracted from the skin of Atlantic salmon (*Salmo salar*). SS-SCP significantly accelerates wound healing rates. This effect is associated with a decrease in proinflammatory cytokines (TNF- α, IL-6 and IL-8) and an increase of anti-inflammatory cytokine IL-10, VEGF and β- FGF [93]. Fish-derived peptides have high biocompatibility and lower allergy risk than mammalian-derived peptides. Large molecular peptides have limited transdermal permeability and thus need to be combined with transdermal delivery technology.

The sea cucumber possesses numerous beneficial effects, including antioxidant, anti-inflammatory, antimicrobial and immunomodulatory activities and the ability to promote wound healing processes [94]. SCCOP is a small molecule oligopeptide derived from fresh sea cucumber through enzymatic hydrolysis [95]. SCCOP has the potential to reduce the expression of CRP, IL-6, IL-8, TNF- α and ROS and meanwhile increases levels of IL-10 and VEGF during the wound healing process in diabetic mice [96]. Multi-target synergy simultaneously regulates inflammation, oxidative stress, immunity and angiogenesis, making it suitable for complex pathological conditions (such as diabetic wounds). The small molecule SCCOP achieves higher absorption efficiency than large molecular peptides, enabling deeper tissue penetration. The sea cucumber farming industry is mature with a stable supply of raw materials. Undoubtedly, these are unique advantages of SCCOP.

#### 3.2.2. Cell migration and proliferation

The chemotactic properties promote cell migration and proliferation, which is an important process in wound healing. Epinecidin-1 stimulates epithelial cell proliferation by modulating cell cycle pathways and promotes migration by regulating extracellular matrix complexes [91]. Another study demonstrated that SCCOP enhances cell proliferation and migration in wound healing by activating the ERK/AKT pathway [97]. SNCP possesses an outstanding capacity to induce human umbilical vein endothelial cells (HUVEC), human immortalized keratinocytes (HaCaT) and human skin fibroblasts (HSF) cells proliferation and migration. It was demonstrated that SNCP can accelerate wound healing and prevent pathological scar formation [98].

#### 3.2.3. Collagen formation and remodelling

During the remodelling period of wound healing, the dermis maintains a dynamic balance between collagen synthesis and degradation, which prevents too much immature collagen to form scars or keloids [99]. Study has shown that SNCP isolated from the peanut worm *Sipunculus nudus* significantly improves wound healing and reduces scar formation in mice. This effect is achieved through regulating collagen formation and remodelling, which enhances collagen deposition and prevent the pathological effects caused by the TGF- β/Smads signalling pathway [100]. Furthermore, SNCP’s role in wound healing may attributed to its small peptides, which improves body absorption. In addition, the essential amino acids in SNCP provide excellent nutritional support for wound healing [98]. Most of these researches using *in vitro*, cell culture and animal models. The scarcity of clinical studies is undoubtedly one of the development directions for SNCP.

TP4, isolated from Nile tilapia (*Oreochromis niloticus*) has antibacterial effects and regulates the innate immune system [89]. In addition, TP4 induces the proliferation of epithelial cells by promoting collagen I, collagen III, keratinocyte growth factor (KGF) and keratin 10. It also enhances antimicrobial and wound healing activities mediated by EGF, TGF and VEGF [101]. Further studies are needed on its long-term safety, optimal administration route (local/systemic) and ways to improve stability *via* genetic engineering or chemical modification. And it is urgent to advance clinical trials to verify human tolerance.

The small molecular polypeptide SMP is prepared from the mantle of *Pinctada martensii* [102]. SMP possesses procoagulant activity. It has been shown to enhance wound closure and inhibit inflammatory response by promoting IL-10 secretion. Additionally, SMP stimulates fibroblasts and keratinocytes secretion, shortens epithelialization time through TGF-β1 and cyclin D1 secretion and accelerates collagen cross-linking deposition [103]. Thus, it is a wound healing agent with great potential and an ideal wound dressing, acting on multiple aspects including inflammation, cell activity and collagen remodelling.

#### 3.2.4. The stimulation of signalling cascades within the cellular environment

**Figure 3.**
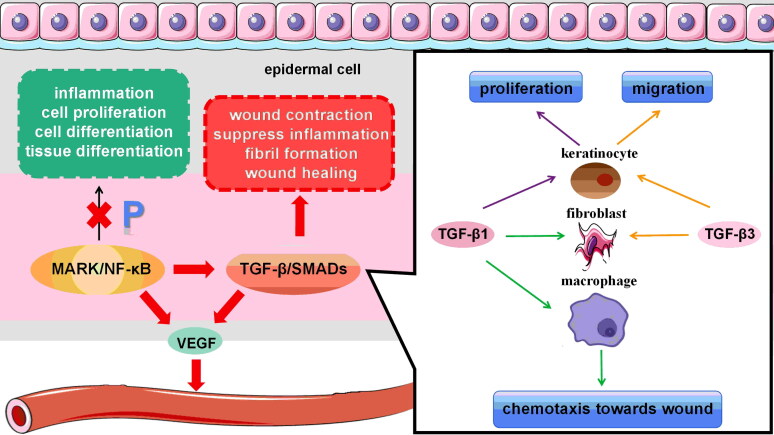
Signalling cascades within the cellular environment in wound healing. MAPK/NF-κB is critical signalling pathways involved in inflammation, cell proliferation and cells and tissues differentiation. The inhibition of their phosphorylation implicates in the facilitation of wound healing. TGF-β1 orchestrates the chemotaxis of macrophages and fibroblasts towards the site of injury, also stimulates keratinocyte proliferation and promotes angiogenesis. TGF-β3 orchestrate the migration of fibroblasts and keratinocytes, thus facilitating re-epithelialization during the wound healing. Activation of the TGF-β/Smads signalling pathway enhances angiogenesis, facilitates wound contraction and suppresses inflammation by promoting fibroblast transformation and integrin expression.

Most wound repair mechanisms are related to the activation of various signalling pathways, such as the Mitogen-activated protein kinase (MAPK)/nuclear factor kappa-light-chain-enhancer of activated B cells (NF- κB) and TGF-β1/Smads signalling pathways [104]. MAPK and NF- κB are two critical signalling pathways involved in inflammation, cell proliferation and differentiation in various cells and tissues. Inhibiting their phosphorylation facilitates cutaneous wound healing. The IGLR extract from *Lamiophlomis rotata* suppresses inflammatory cytokines *via* the RAS/p38 MAPK/NF-κB signalling pathway. It also promotes angiogenesis and fibril formation by increasing VEGF and TGF- β expression in wound tissue during healing [105]. Three highly homologous isoforms of TGF- β, namely TGF- β-1 ∼ 3, play a crucial role in regulating the wound healing processes. It is noteworthy that these isoforms bind to the same receptors during their activation [106]. Inherently, TGF-β1 is widely recognized as a multifaceted gene that plays a pivotal role in directing macrophages and fibroblasts to the injury site during the wound healing acute phase. Additionally, it stimulates keratinocyte proliferation and promotes angiogenesis [107]. TGF-β3 acts as a ligand to direct fibroblast and keratinocyte migration, facilitating re-epithelialization during wound healing [108]. Smads serve as the downstream effector of TGF- β. Activation of the TGF- β/Smads signalling pathway enhances skin angiogenesis, promotes wound contraction and reduces inflammation by stimulating fibroblast transformation and integrin expression (Figure 3) [109].

Given the similarities in wound healing between marine creature and humans, we conjecture that there are more marine bioactive peptides will exist similar effects on humans wound healing. Therefore, these marine bioactive peptide may provide a new direction for the development of wound healing drugs.

### 3.3. Marine bioactive peptides improve wound healing through ceRNA mechanisms using exogenous peptides as molecular probes

The competitive endogenous RNA (ceRNA), which promotes skin wound regeneration through microRNA (miRNA), has also gaining recognition as a valuable molecular tool in wound healing research [110].

**Figure 4.**
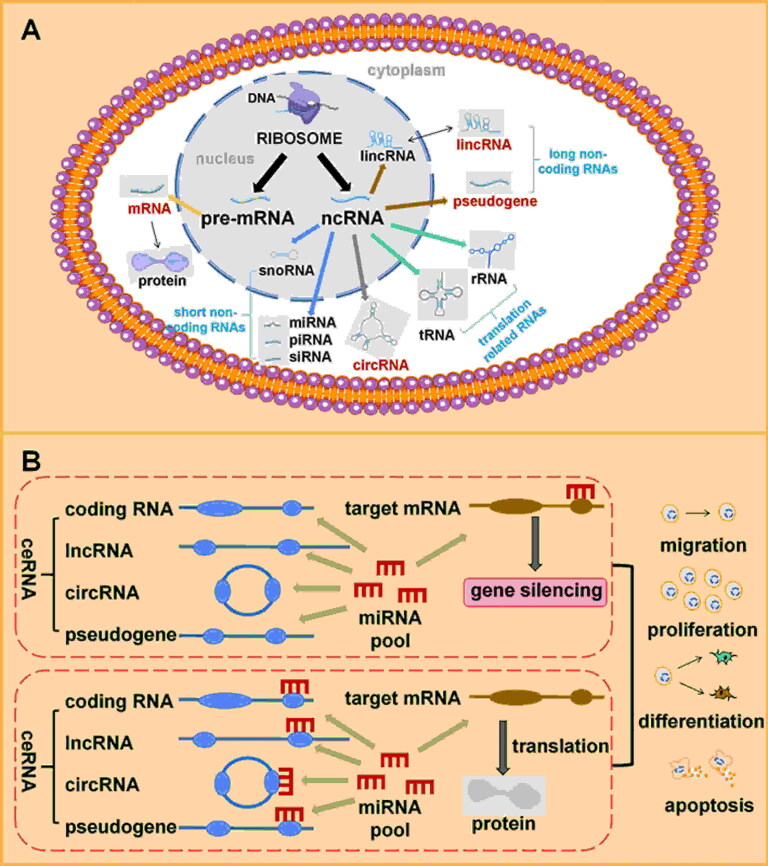
The classification of RNA and the mechanism of ceRNA. (A) RNA is mainly classified into mRNA and ncRNA. ncRNA can be further divided into many types. (B) After miRNAs bind to the target gene, the target gene transfers to a state of gene silencing. But when ceRNA competitively binds to miRNAs, miRNAs are not available to target genes, then target gene expresses to generate proteins.

ceRNAs are RNA molecules, including both coding and non-coding RNAs, that regulate gene expression by competitively binding to shared miRNAs within cells (Figure 4A). These RNA molecules compete for a common pool of miRNAs within the cellular environment [111]. miRNAs are small RNA molecules that regulate gene expression by binding to complementary sequences in target mRNAs, thereby preventing translation or inducing degradation [112]. When multiple RNA molecules have binding sites for the same miRNA, they compete for the limited pool of available miRNAs (Figure 4B) [113]. Recent studies show that miRNAs regulate key biological processes, including cell migration, proliferation, differentiation and apoptosis. Dysregulation of miRNAs is common in various diseases, such as wound healing, and it makes miRNAs potential targets for therapy. For example, miR19a and 20a activate the NF-κB signalling pathway, reducing the production of inflammatory chemokines and cytokines by keratinocytes [114]. The long non-coding RNA (LncRNA) MALAT1-miR-124 network promotes the healing process of subcutaneous adipose tissue induced by hydrogen peroxide (H_2_O_2_) [115]. The combination treatment of Asiaticoside-Nitric Oxide shows that miRNA-21-5p plays a crucial role in promoting wound healing in diabetic rats. Target genes, including TGF-β1, SMAD7 and TIMP3, may contribute to the regulatory mechanisms in diabetic wound healing [116].

In the study of marine organisms, miRNAs regulate antimicrobial and immune processes by directly targeting specific genes. miR-210 and miR-3570 decrease antimicrobial immunity in teleost fish by modulating the RIPK2-mediated NF-κB signalling pathway [117]; miRNA Mc-novel_miR_196 regulates innate immunity in the marine mussel Mytilus coruscus by targeting McTLR-like1, thereby suppressing inflammatory response and apoptosis [118]; miR-144 can enhance p65 activation by inhibiting IκBα, thus impacting the NF-κB signalling pathway in miiuy croaker [119]; in addition, miR-182-3p in the orange-spotted grouper suppresses pro-inflammatory cytokines by regulating the TLR signalling pathway [120].

These discoveries suggest that ceRNAs participate in wound healing mechanisms. They also highlight the potential of marine bioactive peptides as valuable molecular probes to study these complex processes. However, current studies poorly understands the potential of marine bioactive peptides to regulate miRNAs expression and influence signalling pathway activation. These processes may promote re-epithelialization and granulation tissue regeneration, which ultimately accelerate wound healing.

## 4. Marine bioactive peptides combined with novel types of wound dressings

Traditional wound dressings, including gauze, bandages and film coverings, have been used throughout medical history. However, they have shown limited effectiveness in promoting wound healing. Therefore, researchers have sought to enhance efficacy by incorporating bioactive chemicals. These bioactive chemicals in dressings are released through the hydrolysis action of wound enzymes and then delivered to the injury site *via* hydration and diffusion. Due to the rapid absorption of these bioactive chemicals by wound exudate and their inability to effectively penetrate into the wound interior, their efficacy in promoting wound healing is compromised. To address this issue, the combination of hydrogels and nanofibers as wound dressings has been used, enhancing their mechanical properties for clinical use.

**Figure 5.**
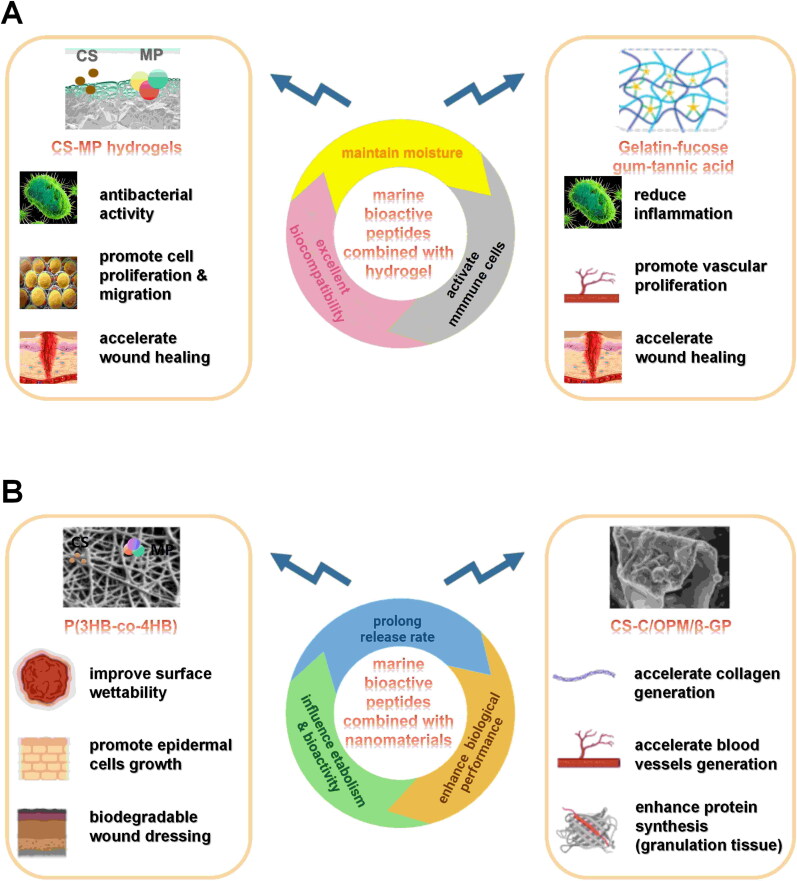
The function of marine bioactive peptides loaded into nanomaterials. The figure lists four new types of wound dressings and their functional mechanisms.

As clinical demands grow, the function of hydrogel wound dressings has evolved from simple physical coverage to multifunctional composites. Some drugs, cytokines, cells and other substances are loaded into the hydrogel: (1) antibacterial hydrogel: loaded antibiotics, inorganic metal NPs, natural or synthetic antibacterial polymers [121]; (2) anti-oxidant hydrogel: loaded natural or synthetic anti-oxidant polymers [122]; (3) anti-inflammatory hydrogel: loaded natural or synthetic anti-inflammatory [123]; (4) hydrogel with controlled delivery characteristics: loaded stem cells [124]; (5) self-healing hydrogel: repair of functional and structural damage by external tension or organizational activities involves dynamic and reversible chemical bonds [125]; (6) stimulus-responsive hydrogel: revision of shape or size to respond to changes in the external stimulus (such as temperature, pH) [126,127]; (7) conductive hydrogel: loaded conductive polymers containing conductivity similar to skin [128] and (8) wound monitoring hydrogel: provision real-time data on wounds (such as wound temperature, bacterial infection and release of antibiotic) [129–131]. Inspired by the excellent properties of hydrogels, researchers have been exploring the incorporation of marine bioactive peptides into hydrogels. Several recent studies have demonstrated their positive effects on wound healing. Ouyang’s research team found that chitosan-marine peptides hydrogels showed antibacterial activity and promoted cell proliferation and migration, well burning healing [132]. Coincidentally, a fucoidan-gelatin (fish skin peptides) hydrogel wound dressing accelerated wound healing by enhancing antibacterial and anti-inflammatory activities (Figure 5A) [133].

The soft nature and high water content of the hydrogel wound dressings provide a microenvironment similar to that of natural ECM, suitable for cell growth. They exhibit moisture maintenance, excellent biocompatibility and immune cells activation, thereby accelerating wound healing [134]. However, they also possess certain limitations, including mechanical fragility and insufficient adhesion properties [135]. To address this issue, nanofibers have been developed to accelerate wound healing. Nanofibers possess a unique structure characterized by hundreds of nanometre-sized networks that create a large surface area and numerous nanoporosity [136]. This microstructure matches the human extracellular matrix, facilitates interactions with cells acting as extracellular matrices, aids in haemostasis, protects against bacterial infections, enables more effective drug binding and enhances the exchange of substances such as oxygen, water, nutrients and metabolic waste. Furthermore, it helps maintain a moist wound environment and good absorption of wound exudate, which is conducive to cell growth, proliferation, adhesion and tissue regeneration [137–139]. Based on the aforementioned points, researchers have combined bioactive peptides with nanofibers to more effectively address wound healing treatment.

Recent studies have demonstrated the efficacy of utilizing nanomaterials, such as polymer nanoparticles, nanostructured lipid carriers, solid lipid nanoparticles and nanoliposomes to encapsulate hydrophobic bioactive peptides [140]. Loading peptides into nanomaterials to achieve continuous release at the application site can significantly enhance therapeutic efficacy. It can protect nanoparticles or chemicals molecules from degradation and overcome the limitations of natural chemicals, including poor solubility, low bioavailability, poor permeability and instability [141].

Furthermore, combining marine bioactive peptides with nanomaterials may influence their metabolism and bioactivity within an organism. For instance, enclosing peptide grades from fish skin gelatin within a nanoliposome system leads to a prolonged release rate; the enhanced biological performance is attributed to the advanced surface chemistry of these systems, their small size and increased surface area [142].

The surface architecture of P(3-hydroxybutyrate-co-4-hydroxybutyrate) [P(3HB-co-4HB)] was enhanced by adding Tilapia fish skin collagen peptides. These peptides were loaded into the nanofibrous P(3HB-co-4HB) construct using a simultaneous dual syringe electrospinning system with eco-friendly solvents [143]. This nano-fibre construct is designed to further improve surface wettability and promote the growth of epidermal cells, while also possessing the desired properties for biodegradable wound dressing [143]. Additionally, the thermosensitive hydrogel chitosan/active oyster peptide microsphere thermosensitive hydrogel (CS-C/OPM/β- GP) was prepared by incorporating active small molecular oligopeptide-oyster peptide (OP) microspheres and utilizing catechol functionalized chitosan as the matrix material (Figure 5B). The nanomaterial can prolong the duration of effect on skin tissue, increase VEGF expression and reduce inflammatory factor levels [144].

Moreover, alginate dressing, chlorhexidine-impregnated paraffin gauze dressing, hydrocolloid dressing, biosynthetic dressing, silver-based dressing, paraffin gauze dressing, polyurethane film and silicone-coated nylon dressing are also widely used in clinical treatment [145].

The marine peptides exhibit unstable bioactivity and are sensitive to external conditions. Therefore, the integration of peptides with composite wound dressings can enhance functionality efficiency of marine bioactive peptides. The skin absorption capacity is related to the molecular weight of drugs. Peptides typically have a large molecular weight and are hydrophilic, which significantly reduces their permeation and absorption through the skin and mucous membranes. However, loading marine bioactive peptides onto wound dressings, such as those prepared with nanotechnology and hydrogels, can address this problem.

However, due to the complexity and dynamic of wound healing process, it is challenging to identify a single dressing that meets the different needs of each phase. For instance, controlling inflammation is crucial during the inflammatory phase of wound healing; meanwhile, providing essential nutrients and cytokines is important during the proliferative and remodelling phases. If a wound dressing contains cytokines or functional components beneficial for the remodelling phase, we cannot exclude potential adverse effects during the inflammatory phase. Therefore, providing functionality on demand represents a promising trend for further research. Ideally, we should develop and refine smart dressings capable of monitoring wound parameters such as pH, temperature and bacterial counts in the wound area. This capability would greatly facilitate both diagnosis and the development of personalized treatment plans for wound healing.

## 5. Advantages and limitations of marine bioactive peptides

Peptides derived from marine resources offer several advantages over those from land animals and other sources. Not only are they plentiful and easily accessible, but there have been few reported toxic effects at effective doses [146]. The peptides from marine organisms are more easily hydrolysed than those from mammals, making them better suited for further processing into peptide derivatives due to their inherent properties [147]. Ziconotide^®^ is a peptide isolated from marine snail Conus magus in 1982 and received the first peptide approval from the US Food and Drug Administration (FDA) for pain medication in 2004 [148]. Seacure^®^ is a commercially available protein supplement for human derived from the fermentation of fish protein. Seacure^®^ has been shown to be effective in mice models *via* the immunomodulatory effects of lactic acid bacteria. Therefore, marine peptides have become a promising area for exploration in the development of new drugs and clinical practice [149].

In addition to its promising safety profile, the utilization of marine bioactive peptides is environmentally friendly. It effectively reduces useful waste that is often discarded by seafood processing industries. This makes them a sustainable and responsible choice for various applications. Furthermore, the skin, scales and bones of fish serve as abundant reservoirs of peptides. Notably, the isolation process does not harm any other organisms [150]. Peptides have a wide range of applications in various skin areas, including but not limited to skin ageing and tissue regeneration. The marine bioactive peptides have demonstrated equivalent efficacy to the currently used antioxidant BHT, emphasizing their potential significance in skincare innovation [151]. Marine bioactive peptides also possesses both structural and functional characteristics that make them inherent substrates for the adhesion, proliferation and differentiation of cells [152].

Marine bioactive peptides exert their biological effects by physically binding to anions and cations on cell membranes or other targets, which reduces drug resistance. However, traditional medications such as antibiotics exert their effects by chemically binding to specific receptors on intracellular or cell membranes, which can easily lead to drug resistance [153]. Marine bioactive peptides also interact with intracellular substances to impact cellular metabolism. These mechanisms not only reduce the development of resistance but also enhance the efficacy of biological activity.

In terms of clinical relevance, marine bioactive peptides demonstrate superior therapeutic efficacy compared to terrestrial plant peptides. The peptides derived from terrestrial plants exhibit several limitations, including poor solubility, low bioavailability and a lack of clinical research. The suboptimal bioavailability of terrestrial plant-derived peptides may be attributed to inadequate absorption and rapid clearance from the body [154]. Synthetic peptides can serve as a substitute to conventional peptide synthesis due to their lower use of toxic chemicals and higher yield. However, their biological activity is significantly inferior to that of naturally extracted peptides, which further enhances the value of natural marine bioactive peptides. Compared to conventional chemical drugs, peptides exhibit relative safety, higher specificity and excellent active efficacy. Their easy excretion from the body makes marine bioactive peptides one of the best options for promoting wound healing [155].

Marine bioactive peptides are plentiful and easily accessible. However, their extraction is often affected by degradation reactions. Peptides experience loss and inactivation at every single step of the extraction process, resulting in ‌comparatively‌ lower yields than other compounds. Additionally, it is important to consider that some marine bioactive peptides derived from protected species or those that are difficult to cultivate cannot be sustainably produced. This will lead to high production costs for marine bioactive peptides. Although artificial synthesis techniques reduce costs, they significantly impair the biological activity of marine peptides. Meanwhile, enzymes, pH and hormone present at the wound area can modify peptides, leading to their deactivation. Consequently, the poor pharmacokinetics of drugs have significantly limited their clinical utility [156]. The instability of natural active peptides is affected by certain factors, including charge number, sequence length, hydrophobicity, specific amino acids and secondary structure [157].

Therefore, when peptides are applied topically to treat skin wounds, it is crucial to investigate their drug metabolism and ability to enhance enzyme tolerance. This is essential for the future development of marine bioactive peptide-based drugs to ensure their effectiveness. Furthermore, an urgent need exists to comprehensively investigate the efficacy and potential adverse effects of marine bioactive peptides on human skin. The current data regarding the safety of natural marine bioactive peptides is still not sufficiently comprehensive. Moreover, marine bioactive peptides are not completely non-toxic. They share many characteristics with eukaryotic signalling peptides and can be internalized by cells, causing toxicity such as mast cell degranulation, cell apoptosis, or extracellular DNA transfer. Whether a peptide has haemolysis depends on the structure of the N-terminal domain. Therefore, the assessment of the haemolysis of marine bioactive peptides may be approached by examining their secondary structure.

**Figure 6.**
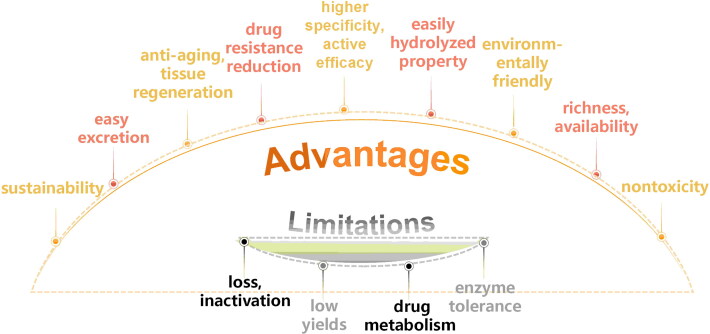
The advantage and limitations of marine bioactive peptide.

The deficiencies mentioned above limit the development of marine bioactive peptides, making them insufficient for large-scale production demands. Although marine bioactive peptides have some limitations, their wide range of benefits far outweighs these drawbacks (Figure 6).

## 6. Conclusions and future perspectives

Marine resources are deemed a crucial source of peptides in academic research [158]. These bioactive peptides are anticipated to emerge as novel drug candidates for the treatment of various conditions. Despite the extensive exploration of bioactive peptides for wound healing, with a particular focus on their potential for drug development, research and investigation into marine-derived peptides remain constrained. There is immense potential within this resource pool that has yet to be fully realized. Currently, there are 20 ongoing clinical trials involving peptide-based drugs, and over 400 new peptide drugs are developed annually [159].

Nowadays, many substantial experimental research has demonstrated the beneficial effects of marine bioactive peptides in both acute and chronic wound healing. These effects include promoting cell proliferation and migration, enhancing angiogenesis and exerting anti-inflammatory actions. However, in chronic wounds, the promotion of ECM remodelling, re-epithelialization, immune regulation, improvement of microenvironmental defects and repair mechanisms become more pronounced and exhibit distinct characteristics compared to acute wounds. There is a notable lack of in-depth exploration regarding mechanisms and molecular targets. The underlying mechanisms of wound healing may offer valuable insights. Currently, the most common signalling pathways associated with wound healing include the macrophage polarization (inflammatory phase) and TGF-β1/SMAD signalling pathway (proliferative phase) [160]. Further research is required to explore the potential mechanisms and molecular targets of marine bioactive peptides in wound healing, which will enhance recognition of their clinical application value. Marine bioactive peptides have been extensively studied in skin wound research, current investigations are still not comprehensive. For instance, the number of experiments remains limited, and there is a lack of exploration of the relationship between dosage and side effects, as well as deficiencies in randomized controlled trials. Basic experiments form the theoretical basis for clinical applications, and this aspect cannot be ignored.

Marine bioactive peptides have potential topical and oral applications for wound healing. They exhibit significant biological activities, such as modulating inflammatory response, oxidative stress and abnormal expression of matrix metalloproteinase and chemotactic factors. Therefore, researchers must further investigate the long-term safety and formulations of these products to ensure successful commercialization. In addition, developing complementary products is essential to improve the accessibility and efficiency of marine bioactive peptides. This will enhance their potential for tissue regeneration during wound healing. However, peptides are susceptible to enzymatic degradation, rapid clearance, short half-life and instability of biological activity [159]. In the future, the pharmacokinetic properties of marine bioactive peptides can be improved by increasing cell membrane binding site, enhancing enzyme metabolic stability, extending biological activity and altering excretion rates. The journey towards the clinical application of peptides remains extensive and fraught with challenges.

The process of peptide acquisition from marine sources also requires optimization. By reducing peptide loss at each step, production costs can be lowered. Lowering costs benefits the future widespread application of clinical practices and helps provide patients with affordable treatment options. With the advancement of molecular biology, the expression of peptides in plants using the gene transformation has become a matured technology [161]. This method inspires us to apply gene transformation technology to produce marine bioactive peptides.

The successful application of marine bioactive peptides in emerging wound dressings will lead to the development of effective formulas, including hydrogels, nanomaterials and 3D bioprinting techniques, etc. It is notable that various stabilizers such as surfactants, polymers and covalent adsorption bonding should be used in developing wound dressings to reduce skin irritation. Moreover, standardized and controllable models are needed to accurately represent the healing mechanisms of various wound types. The requirements of ideal real-time monitoring indicators should be suitable for assessing wound conditions. They should also support the design of personalized treatment plans. Most pH response of wound dressings is more friendly to acidic pH, but the pH of chronic wounds is alkaline. This contrast highlights the need to develop wound dressings specifically designed for alkaline environments, representing a promising new direction in wound care.

**Figure 7.**
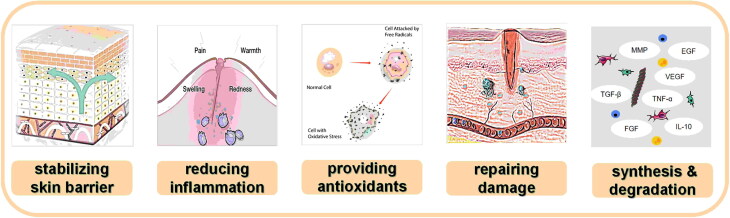
Marine bioactive peptides have various benefits.

In conclusion, researchers can obtain marine bioactive peptides with potent antibacterial and skin repairing properties. These versatile peptides have a wide range of applications, including external application, oral intake and as dietary supplements. They offer numerous benefits such as stabilizing the skin barrier, reducing inflammation, providing antioxidants, repairing damage, promoting synthesis and delaying degradation (Figure 7). Marine bioactive peptides, regarded as natural and healthy, can attract consumers and drive social momentum. This offers valuable insights for future research in this field and establishes a solid foundation for the global wound healing molecules market.

## Data Availability

No data were generated in this work. This review uses data from several databases, including ScienceDirect, Google Scholar, Scopus, PubMed and Springer Link.
